# Alcohol discrimination and preferences in two species of nectar-feeding primate

**DOI:** 10.1098/rsos.160217

**Published:** 2016-07-20

**Authors:** Samuel R. Gochman, Michael B. Brown, Nathaniel J. Dominy

**Affiliations:** 1 Department of Biological Sciences, Dartmouth College, Hanover, NH 03755, USA; 2 Department of Anthropology, Dartmouth College, Hanover, NH 03755, USA

**Keywords:** dietary ethanol, alcohol dehydrogenase class IV (ADH4), *Daubentonia madagascariensis*, *Nycticebus coucang*

## Abstract

Recent reports suggest that dietary ethanol, or alcohol, is a supplemental source of calories for some primates. For example, slow lorises (*Nycticebus coucang*) consume fermented nectars with a mean alcohol concentration of 0.6% (range: 0.0–3.8%). A similar behaviour is hypothesized for aye-ayes (*Daubentonia madagascariensis*) based on a single point mutation (A294V) in the gene that encodes alcohol dehydrogenase class IV (ADH4), the first enzyme to catabolize alcohol during digestion. The mutation increases catalytic efficiency 40-fold and may confer a selective advantage to aye-ayes that consume the nectar of *Ravenala madagascariensis*. It is uncertain, however, whether alcohol exists in this nectar or whether alcohol is preferred or merely tolerated by nectarivorous primates. Here, we report the results of a multiple-choice food preference experiment with two aye-ayes and a slow loris. We conducted observer-blind trials with randomized, serial dilutions of ethanol (0–5%) in a standard array of nectar-simulating sucrose solutions. We found that both species can discriminate varying concentrations of alcohol; and further, that both species prefer the highest available concentrations. These results bolster the hypothesized adaptive function of the A294V mutation in ADH4, and a connection with fermented foods, both in aye-ayes and the last common ancestor of African apes and humans.

## 1. Background

Mounting evidence suggests that dietary ethanol, or alcohol, is a prevalent component in the natural diets of non-human primates [1–9]. This premise, which is contested [10], is supported by recent findings concerning alcohol dehydrogenase class IV (ADH4) [11], the first enzyme to encounter and catabolize alcohol during digestion [12]. Carrigan *et al.* [11] reported the protein sequences and corresponding kinetic activities of ADH4 in 18 primate species and resurrected nine ancestral proteins to better understand the evolution and functional ecology of ADH4. This innovative approach revealed three key findings. First, ADH4 is practically inactive toward ethanol in most primate species. Second, a single amino acid change (A294V) in some species resulted in a 40-fold increase in ethanol oxidation, substantially improving enzymatic efficiency. Third, this mutation arose independently in two distantly related primates, the aye-aye (*Daubentonia madagascariensis*) and the last common ancestor of African apes and humans.

Carrigan *et al.* [11] proposed that increasing terrestrial travel exposed the last common ancestor of African apes and humans to fermented fruits on the forest floor; and further, that consuming such fruits favoured retention of the A294V mutation. This adaptive hypothesis is alluring [13] but some observers have inferred or imputed a primate aversion to fermented resources [10]. At the same time, chimpanzees are known to ingest the fermented exudates of some plants [8,14]. For example, the chimpanzees of Bossou, Guinea consume sap from the raffia palm (*Raphia hookeri*) with a mean alcohol concentration of 3.1% (range: 0.6–6.9% [8]). This behaviour is somewhat unnatural (the chimpanzees capitalize on the presence of sap-collecting plastic containers [8]), but it indicates a proclivity for alcohol and is therefore compatible with the adaptive hypothesis of Carrigan *et al.* [11]. Still, the problems with evaluating this hypothesis are twofold. First, the alcohol content of overripe or fermented fruits in African forests, or the attraction of chimpanzees to them, await systematic study. Second, the aye-aye (*D. madagascariensis*) is an exemplar of dietary specialization that poses a challenge to any adaptive interpretation of the A294V mutation.

### 1.1. Aye-aye (*Daubentonia madagascariensis*)

**Figure 1.**
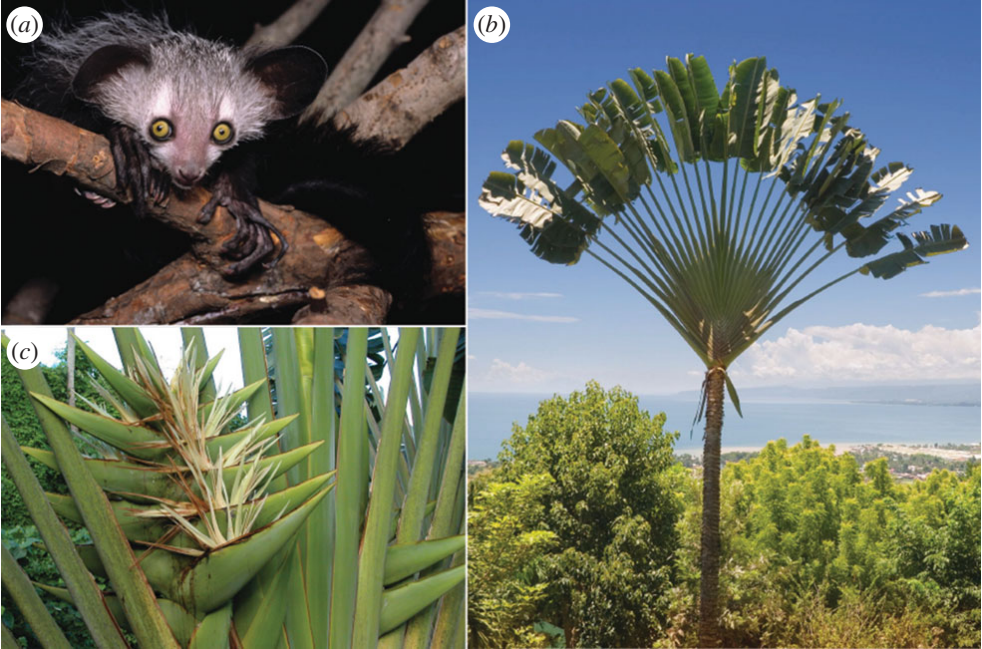
Natural history underlying the hypothesized adaptive function of the A294V mutation in the gene that encodes ADH4 [11]: (*a*) the aye-aye (*Daubentonia madagascariensis*; photograph by David Haring, reproduced with permission); (*b*) the traveller's tree (*Ravenala madagascariensis*; Strelitziaceae) is an enduring symbol of Madagascar [15] in part because of its distinctive, distichous leaf arrangement; (*c*) the axillary inflorescence of *R. madagascariensis* is distinguished by congested overlapping bracts, some of which are shown with protruding flowers (photograph by Gerald McCormack, reproduced with permission)*.* For aye-ayes, the sturdy bracts and flowers of *R. madagascariensis* are potential sources of fermented nectar, perhaps, especially areas of abundant stands (‘*Ravenala* forests’ [16]) in eastern Madagascar.

The aye-aye is a nocturnal lemur endemic to Madagascar (figure 1*a*). It is an enduring source of fascination, both because of its many idiosyncrasies and because it is the sole surviving member of a lineage with an origin approximately 70 million years ago [17]. *Daubentonia* is therefore allocated to its own family (Daubentoniidae) and infraorder (Chiromyiformes). The aye-aye is perhaps best known for its acoustic foraging behaviours, termed percussive foraging or tap-scanning [18], and suite of anatomical specializations, particularly in the hand, skull and central nervous system [18–27]. The elongated middle finger of aye-ayes—described as villiform, filamentous, and grotesquely attenuated—is one of its most outstanding traits, for it is equipped with a unique ball-and-socket metacarpophalangeal joint [24] and capable of extreme mobility [28] and speed (tap intervals of 97.7 ± 19.9 ms [29]). The central function of this extraordinary instrument is to detect, localize and extract the larvae of longhorn (cerambycid) beetles in decaying wood [30].

Given that larvae are an improbable source of alcohol, it is tempting to interpret the A294V transition of aye-ayes as a spurious mutation. However, aye-ayes devote as much as 20% of wet-season feeding time to the nectar of *Ravenala madagascariensis* [31], the traveller's tree (figure 1*b*). Aye-ayes use their middle digits to probe for and withdraw nectar, an action that strongly suggests effective pollination [32]. The sucrose content of the nectar ranges from 11.5 to 15.5%, with mean published values of 12.2 ± 1.3, 14.2 and 14.5% [31–33]. Perhaps significantly, the large bracts of *R. madagascariensis* (figure 1*c*) contain another liquid described as ‘slimy … copious and always readily available, (with a) low sugar content reward of 2.8%’ [33]. This description resembles accounts of fermented nectar [34], but the extrafloral location and low sugar content is puzzling, suggesting a distinct exudate. Alternatively, the bracts may accumulate excess or spilled nectar, the sugar of which is diluted by rain or consumed by yeast during fermentation. Access to one or both liquids raises the possibility that aye-ayes consume non-trivial quantities of alcohol on a seasonal basis. Although speculative, this hypothesis [11] agrees well with observations of another nocturnal primate, the slow loris (*Nycticebus coucang*).

### 1.2. Slow loris (*Nycticebus coucang*)

**Figure 2.**
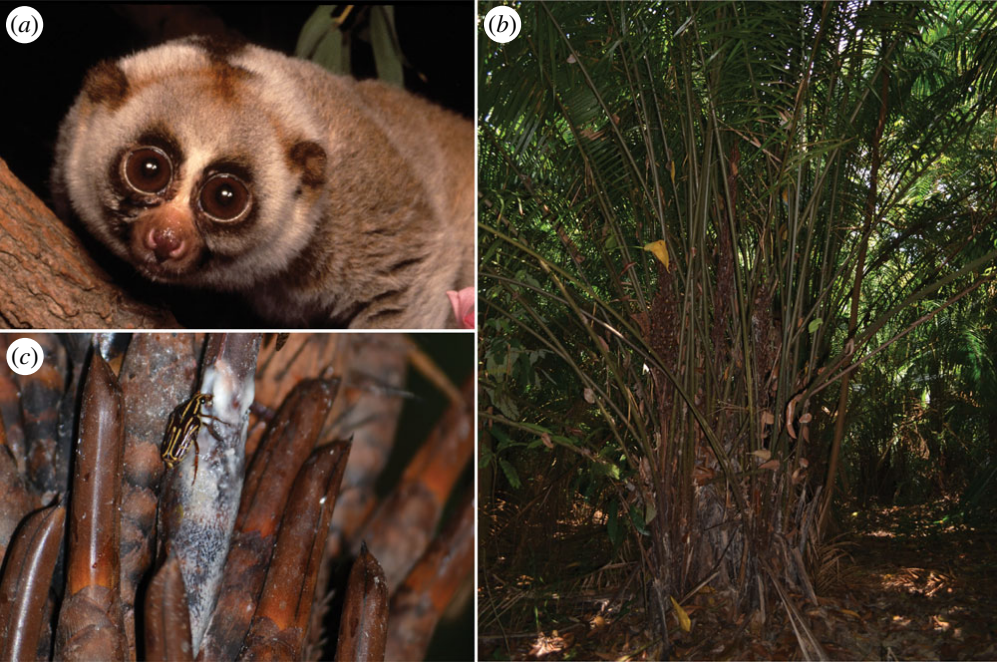
At least one primate species is a verified consumer of fermented nectar [34]: (*a*) the slow loris (*Nycticebus coucang*; photograph by David Haring, reproduced with permission); (*b*) the bertam palm (*Eugeissona tristis*; Arecaceae) is a fiercely spiny acaulescent palm endemic to Peninsular Malaysia and Thailand [35] (photograph by Annette Zitzmann, reproduced with permission); (*c*) the woody pencil-shaped flowers of *E. tristis* are well suited for supporting the weight of scansorial mammals attracted to the frothing, fermented nectar [36]. The presence of a nitidulid beetle demonstrates the attraction of insects to fermented nectar; it is also a potential vector of inoculation with yeast [34] (photograph by Annette Zitzmann, reproduced with permission).

The slow loris is a nocturnal lorisoid primate endemic to Southeast Asia (figure 2*a*). In Perak, Malaysia, a substantial proportion of its diet (41% of feeding observations) is focused on the fermented nectar of the bertam palm (*Eugeissona tristis*) (figure 2*b,c* [34,37]). The nectar of *E. tristis* contains sucrose (approx. 10% [37]) and alcohol concentrations as high as 3.8% (vol/vol) (mean and median levels = 0.6 and 0.5%, respectively [34]). Nightly estimates of alcohol consumption suggest that slow lorises are unlikely to suffer intoxication [34], but they do highlight a potential selective pressure on ADH4 and other members of the ADH family. At present, however, the protein sequence and corresponding kinetic activity of ADH4 are unknown for slow lorises.

### 1.3. Motivation and study design

Recent findings concerning aye-ayes and slow lorises point to habitual interactions with fermented nectar, and it follows that natural selection would have favoured a discriminating aversion or preference for low concentrations of alcohol. Here, we describe and report the results of a multiple-choice feeding experiment designed to test this basic premise.

## 2. Multiple-choice feeding experiment

### 2.1. Animal subjects and study site

We studied two aye-ayes at the Duke Lemur Center (DLC), Durham, NC, USA; an adult female (‘Morticia’; mass: 2.70 kg), born in the wild in 1988, and an adult male (‘Merlin’; mass: 2.96 kg), born at the DLC in 1994. Morticia was captured in 1991 near Anjimangirana, northwest Madagascar [38], an area without formal descriptions of *Ravenala* [16]. We also studied an adult slow loris, a female (‘Dharma’; mass: 1.06 kg), born at the DLC in 2000. The diets of both species contain chow gruel, mealworms and a medley of ripe fruit, which is expected to contain trace amounts of alcohol. Lifetime exposure to alcohol is therefore limited. The study animals were housed separately in temperature-controlled enclosures and maintained on a reverse light cycle. Our experiments (between 10.30 and 15.00) were conducted during the wake cycles of the animals and therefore under conditions of semidarkness.

### 2.2. Experimental design

We modified a resin outdoor dining table (Truva; height: 29 inch [74 cm]; diameter: 42 inches [107 cm]) to create a sturdy, sanitizable experimental array (electronic supplementary material, figure S1*a*). The circular array consisted of eight 1 oz [29.6 ml] containers recessed into the table surface. A 0.5 inch [1.27 cm] hole was drilled into the 2 inch [5.1 cm] cap of each container to enable olfactory assessment and digital access, while limiting evaporation (electronic supplementary material, figure S1*b*).

To simulate the typical nectars of *R. madagascariensis* (germane to aye-ayes) and *E. tristis* (germane to slow lorises), we created stock solutions of 14% and 10% sucrose by volume, respectively [31–34,37]. To simulate fermented nectars, we used these stock solutions to create serial dilutions of pure (99.5%) ethanol (Fisher catalogue no. BP2818500). This protocol has the practical advantage of simplifying the daily on-site production of fresh solutions, but it has the disadvantage of systematic imprecision. For instance, our serial dilutions of 1.0%, 3.0% and 5.0% ethanol in a solution of 14% sucrose are in fact dilutions of 0.995%, 2.985% and 4.975% ethanol in solutions of 13.86%, 13.58% and 13.30% sucrose, respectively. Similarly, our serial dilutions of 1.0%, 2.0% and 4.0% ethanol in a solution of 10% sucrose are in fact dilutions of 0.995%, 1.990% and 3.980% ethanol in solutions of 9.9%, 9.8% and 9.6% sucrose, respectively. We held the volume of solution in each container constant at 25 ml (aye-ayes) or 10 ml (slow loris), a difference that is proportional to the body masses of our study animals.

For the two aye-ayes, the experimental array contained tap water and four 14%-sucrose solutions with varying concentrations of ethanol: 0.0 (control), 1.0, 3.0 and 5.0%, a range that corresponds with recorded variation in primate-edible fruits and exudates [2–9,34]. To avoid animal habituation, the positions of the five containers in the array were randomized at the start of each trial; and to avoid observational bias, all behavioural data were collected blind to the contents of individual containers. Both aye-ayes were inquisitive animals and they themselves initiated most trials by mounting the table and entering the array voluntarily. A trial was defined by the onset and offset of physical interactions with individual containers. The trials ended when direct contact with the array ceased for 5 min. Some trials were terminated when the animals gnawed the apparatus instead of probing the containers, indicating satiety or disinterest in the experiment.

To test whether aye-ayes demonstrate a preference for controls or experimental treatments, we quantified probative behaviours as a proxy for motivation. This behaviour entailed three discrete actions: (i) extension of the middle finger, (ii) probing of individual containers and (iii) finger withdrawal coupled with immediate oral intake. This stereotypical sequence, or digital-probe-to-mouth-event (DPME), is rapid and difficult to quantify with real-time visual observations. Accordingly, we filmed all trials with a digital video camera equipped with nightshot capabilities (Sony AVCHD HDR-CX405; electronic supplementary material, video S1). For each variable, we measured DPMEs and calculated the ingested mass by subtraction. For each aye-aye, we performed one trial per day over 15 days for a total of 30 trials.

For the slow loris, the experimental array contained tap water and four 10%-sucrose solutions of 0.0 (control), 1.0, 2.0 and 4.0% ethanol, a range that corresponds with recorded variation in the nectar of bertam palms (*E. tristis*) [34]. Trial conditions were identical to those of the aye-ayes with two exceptions: (i) the slow loris was induced to the centre of the array with raisins or honey and (ii) the lids were removed from the containers to allow direct oral access to each solution (electronic supplementary material, figure S1*c*). To test whether our study animal demonstrated a preference for individual variables, we calculated the ingested mass of each solution by subtraction. We performed one trial per day over 5 days for a total of five trials.

### 2.3. Data analysis

For aye-ayes, the number of DPMEs and consumed masses varied considerably across the 30 trials. Accordingly, we calculated proportional consumption by dividing the number of DPMEs or mass consumed for each variable by the corresponding totals for each trial [39]. To test the null hypothesis (i.e. no differential consumption between solutions), we used a multivariate Hotelling's *T*^2^ test [39,40]. To test whether aye-ayes also demonstrated a preference for alcohol, we performed a post hoc comparison of lower (0.0 and 1.0%) and higher (3.0 and 5.0%) concentrations of alcohol using repeated-measures ANOVA [40]. For the slow loris, the mass of liquid consumed varied somewhat across the five trials. We therefore replicated the preceding statistical analyses, except that our post hoc comparison of lower (0.0 and 1.0%) and higher (2.0 and 4.0%) concentrations of alcohol differed per experimental conditions. Statistical procedures were performed in JMP v. 12.1.0 (SAS Institute, Cary, NC, USA) and G*Power v. 3.1.9.2 [41]; all probability levels are two-tailed, with significance at *α* < 0.05.

## 3. Results

### 3.1. Aye-aye (*Daubentonia madagascariensis*)

Covariation of the two measures, digital-probe-to-mouth-events (DPMEs) and mass consumed, was nonlinear but highly predictive across 30 trials (*R*^2^ = 0.96; electronic supplementary material, figure S2), indicating that error from spillage or miscounted DPMEs was distributed evenly through the experiment.

The aye-ayes discriminated between conditions regardless of whether tap water was included (Hotelling's *T*^2^ = 1267.3, *F* = 218.5, *p* < 0.0001) or excluded (Hotelling's *T*^2^ = 41.8, *F* = 9.4, *p* < 0.0001) as a control variable. We obtained similar results when we analysed consumption by mass (water included: Hotelling's *T*^2^ = 7100.7, *F* = 1217.2, *p* < 0.0001; water excluded: Hotelling's *T*^2^ > 9000.0, *F* > 2000.0, *p* < 0.0001), suggesting that aye-ayes can readily detect varying concentrations of alcohol and adjust their behaviours accordingly.

**Figure 3.**
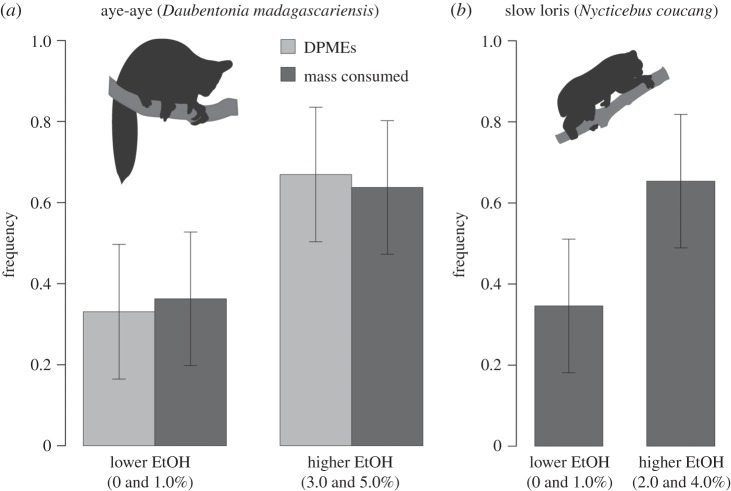
Bar graphs of mean proportional alcohol consumption (whiskers: ± 1 s.d.) across all trials in a multiple-choice feeding experiment: (*a*) for aye-ayes (*n* = 2 animals, 15 trials each), the mean proportion of digital-probe-to-mouth-events (DPMEs) and the consumed mass of alcohol in an array of *Ravenala* nectar-simulating solutions differed between lower- (0.0 and 1.0%) and higher-alcohol (3.0 and 5.0%) concentrations, indicating a preference for higher concentrations; (*b*) for a slow loris (*n* = 1 animal, 5 trials), the consumed mass of alcohol in an array of *Eugeissona* nectar-simulating solutions differed significantly between lower- (0.0 and 1.0%) and higher-alcohol (2.0 and 4.0%) concentrations, indicating a preference for higher concentrations.

To test whether aye-ayes demonstrate a preference for alcohol, we performed a post hoc comparison of lower (0.0 and 1.0%) and higher (3.0 and 5.0%) concentrations and detected a preference for higher concentrations, as measured by DPMEs (repeated-measures ANOVA: *F* = 12.95; *p* < 0.0001) and mass consumed (repeated-measures ANOVA: *F* = 18.87; *p* < 0.0001; figure 3*a*). Another indication of preference is indirect but telling. Containers with higher-alcohol concentrations elicited many scores of DPMEs even after the liquid content was emptied (see the inflection at 25 ml; electronic supplementary material, figure S2). Compulsive digital probing for residual traces of alcohol suggests a strong attraction or craving.

### 3.2. Slow loris (*Nycticebus coucang*)

The drinking behaviour of a slow loris, as measured by the proportion of mass consumed, varied significantly across five trials when water was included as a control variable (Hotelling's *T*^2^ > 9000.0, *p* < 0.0001), but not when it was excluded (Hotelling's *T*^2^ = 508.9, *F* = 31.8, *p* = 0.13). These conflicting findings reflect (i) a relative aversion to tap water and (ii) a limited sample of five trials, respectively. A post hoc power analysis of the latter result [effect size: Mahalanobis distance, *D*^2^ = 19.0; power (1 − *β*) = 0.84; *α* = 0.05, two-tailed] indicates that an additional (sixth) trial would have resulted in statistical significance. To test whether the slow loris preferred alcohol, we performed a post hoc comparison of lower (0.0 and 1.0%) and higher (2.0 and 4.0%) concentrations and detected a preference for higher concentrations (repeated-measures ANOVA: *F* = 7.55; *p* < 0.03; figure 3*b*).

## 4. Discussion

Our multiple-choice preference experiments faced several constraints, namely a limited number of individuals and trials, resulting in a small overall sample size. This weakness is compensated, perhaps by the statistical strength and novelty of our findings. We found that members of two species of nectivorous primate—the aye-aye (*D. madagascariensis*) and slow loris (*N. coucang*)—can discriminate between varying concentrations of alcohol in nectar-simulating solutions; and further, that both species prefer the highest concentrations of alcohol available to them. This result bolsters the hypothesized adaptive function of the A294V mutation in the gene that encodes ADH4 in aye-ayes [11], but it also calls attention to several empirical voids.

First, the flowers and bracts of *R. madagascariensis* (figure 1*c*) are uncertain sources of fermented nectar. The potential for alcohol in these nectariferous tissues is a topic that invites immediate attention. Second, ADH4 is unstudied in slow lorises, the only verified consumer of fermented nectar among primates [34]. The protein sequences and kinetic activities of ADH and aldehyde dehydrogenase (ALDH) enzymes in this species could prove instructive. Third, our experiment suffers from limited sampling within the primate phylogeny, both at the molecular and behavioural levels. It is unknown whether nectarivorous strepsirrhines lacking the A294V mutation—such as the southern lesser galago (*Galago moholi*) and grey mouse lemur (*Microcebus murinus*) [11]—would perform any differently in our experiment. A similar preference for alcohol is perhaps likely given that several species of monkey are attracted to alcohol despite lacking the A294V mutation (e.g. baboons, vervets, macaques; review: [6]), suggesting that the A294V mutation might be irrelevant if compensation exists elsewhere in the alcohol metabolic pathway. ADH4 is merely one of many enzymes involved in alcohol metabolism.

Setting these inconvenient facts aside, it is tempting to suggest that our results are germane to human evolution. A preference for alcohol among aye-ayes is compatible with the hypothesis advanced by Carrigan *et al.* [11], i.e. that the presence of the A294V mutation in a lineage is plausible evidence of fermented foods in the diet. It follows that the last common ancestor of African apes and humans consumed fermented foods, but the nature of these foods is debated. Carrigan *et al.* [11] suggested fermented fruits on the forest floor were the vehicle for alcohol in the diet, but fermented exudates are another possibility [8]. A problem with any of these ideas is that systematic data are scarce, in part because alcohol was once viewed as a deterrent to vertebrate frugivores [42] and because wild primates show few outward signs of intoxication [10]. In consequence, the chronic consumption of alcohol at low levels in ripe and overripe fruits, as originally emphasized by Dudley [1], remains an underappreciated factor in the foraging ecology of primates, and alcohol is seldom measured in primate foods.

Recent evidence, however, indicates that low concentrations of alcohol are prevalent in fruits [2–5,9] and exudates [8,34], although samples sizes are small. Still, it is becoming increasingly likely that many or most primates habitually consume alcohol. Future research on the evolutionary ecology of yeast--plant--primate interactions promises to be rewarding, a prospect anticipated by Corner [43] who, decades ago, observed:

Far too little seems to be known about tropical yeasts, or, if so, the knowledge has not filtered out of scientific journals. Many an exudate from the broken limbs of trees, broken climbers, and the broken leaf-stalks and flowering stalks of palms is fermented by wild yeasts in the forest. My botanical monkeys so relished these slimy and alcoholic clots that I concluded that primitive man must have known about them and that mankind could never have been teetotal until recently. [p. 138]

## Supplementary Material

Figure S1

## Supplementary Material

Figure S2

## Data accessibility

All data were deposited in the Dryad Digital Repository (http://dx.doi.org/10.5061/dryad.831g4).
