# Supplementary figures and images for: Alcohol discrimination and preferences in two species of nectar-feeding primate
Source: R Soc Open Sci. 2016 Jul 20;3(7):160217. doi: 10.1098/rsos.160217 (PMC4968469; doi:10.1098/rsos.160217)

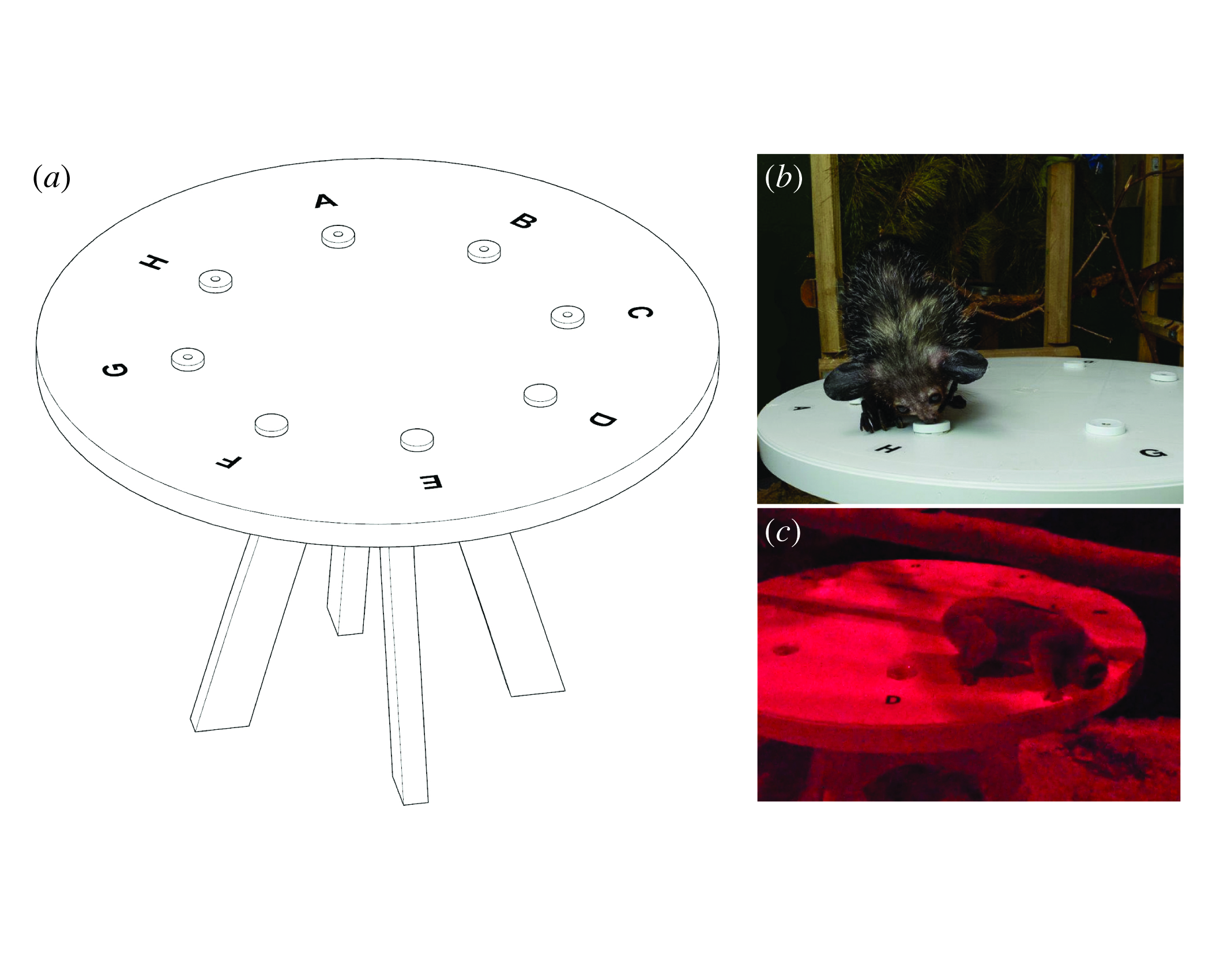

Supplement: Figure S1 [file rsos160217supp1.tif]

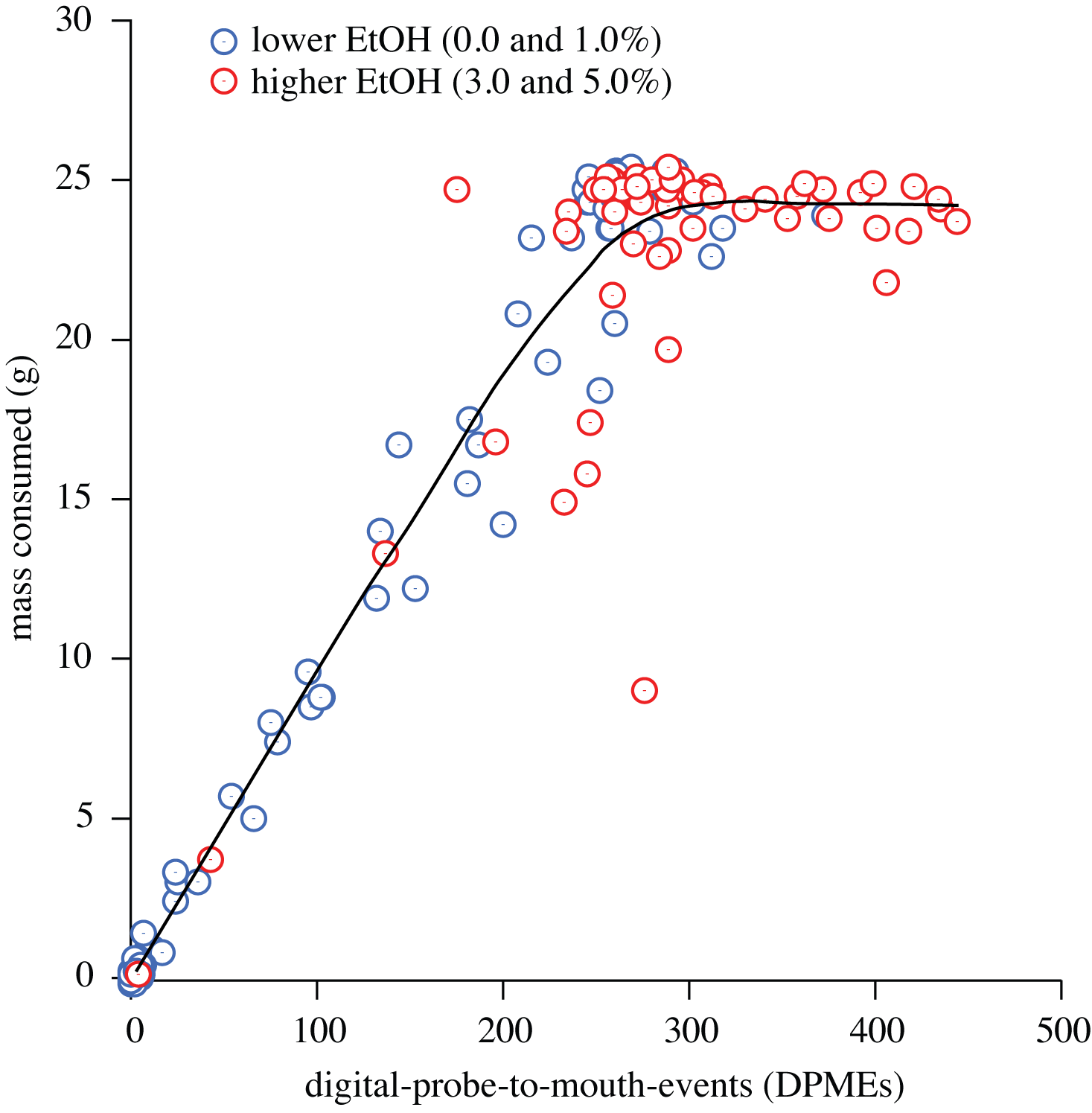

Supplement: Figure S2 [file rsos160217supp2.tif]
